# Glia selectively approach synapses on thin dendritic spines

**DOI:** 10.1098/rstb.2014.0047

**Published:** 2014-10-19

**Authors:** Nikolai Medvedev, Victor Popov, Christian Henneberger, Igor Kraev, Dmitri A. Rusakov, Michael G. Stewart

**Affiliations:** 1 Department of Life and Health Sciences, The Open University, Milton Keynes MK7 6AA, UK; 2 Institute of Cell Biophysics, Russian Academy of Sciences, Pushchino 142290, Russia; 3 Institute of Neurology, University College London, Queen Square, London WC1N 3BG, UK; 4 Institute of Cellular Neurosciences, University of Bonn Medical School, Bonn, Germany

**Keywords:** glia protection, synapses, thin spines

## Abstract

This paper examines the relationship between the morphological modality of 189 dendritic spines and the surrounding astroglia using full three-dimensional reconstructions of neuropil fragments. An integrative measure of three-dimensional glial coverage confirms that thin spine postsynaptic densities are more tightly surrounded by glia. This distinction suggests that diffusion-dependent synapse–glia communication near ‘learning’ synapses (associated with thin spines) could be stronger than that near ‘memory’ synapses (associated with larger spines).

## 1. Introduction

In the hippocampus, high-affinity transporters populating astroglia membranes rapidly buffer the excitatory neurotransmitter glutamate released by synaptic discharges [1–4]. This powerful uptake system maintains low ambient extracellular glutamate (20–30 nM), providing a ‘silent’ background for transient excitatory signals [5]. However, astrocytes occupy less than 10% of tissue volume in area CA1 of the hippocampus [6], and synchronous synaptic releases may give rise to extra- or inter-synaptic actions of glutamate [7–9]. Indeed, astrocyte protrusions occur unevenly in the hippocampal neuropil, closely approaching only 20–30% of excitatory synapses [10], with no apparent relationship to the morphology of the host dendritic spines [11]. However, glial coverage of synapses varies depending on the organism's physiological state [12,13], which raises the question as to whether there is an adaptive significance for the uneven distribution of astroglia near hippocampal synapses.

To investigate this, we first documented the architectonics and the tissue volume fraction occupied by live astrocytes in dentate neuropil and, second, reconstructed in three dimensions the ultrastructure of contiguous astrocyte fragments together with the adjacent excitatory synapses. To quantify juxtaposition of synapses and astroglia, we first calculated the shortest distances between astrocyte membranes and the nearest edges of postsynaptic densities (PSDs) at 136 ‘thin’ and 53 ‘mushroom’ dendritic spines [14,15] of dentate granule cells. Second, we applied an integrative weighted-distance measure providing cumulative data about spatial glial coverage of PSDs. We found that glial membranes occur substantially closer to the PSDs of thin dendritic spines compared with mushroom spines. Because mushroom and thin dendritic spines have been associated with the different dominant synaptic receptors [16–18] and different stages of synaptic plasticity [19–21] (although see [22]), the results suggest that glia may selectively approach synapses that are in the process of physiological ‘learning’.

## 2. Material and methods

### (a) Two-photon microscopy and morphometry

**Figure 1.**
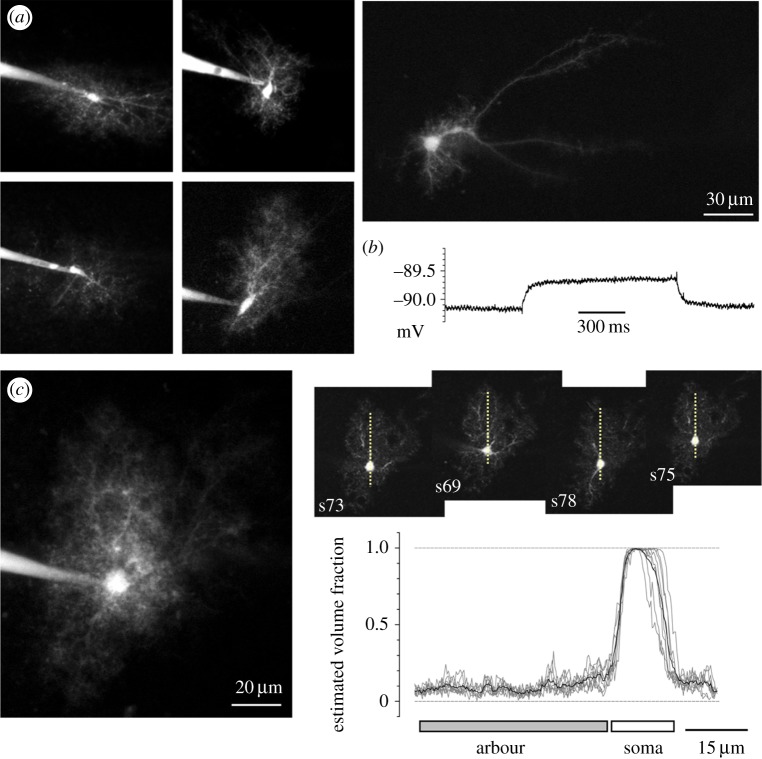
Morphology of live astrocytes in dentate gyrus. (*a*) Characteristic morphologies of individual astrocytes held in whole-cell mode and filled with Alexa Fluor 594 (*λ_x_* = 800 nm); each fluorescence image represents an averaged *Z*-stack of 70–110 consecutive *X–Y* two-photon excitation sections taken with 0.5 µm *Z*-axis steps; patch pipettes are seen. Image on far right, an astrocyte representing a sub-group with prominent dendritic trunks [24]. (*b*) Input resistance measurement in a typical ‘passive’ astrocyte; whole-cell current clamp mode, a voltage response to a 200 pA current injection pulse is shown (upper trace; also see text and electronic supplementary material, figure S1). (*c*) In each astrocyte, distribution of the tissue volume fraction 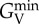 occupied by thin, indicator-filled astrocyte processes (excluding areas containing large dendritic trunks) is measured in a *Z*-series of thin *X–Y* optical sections provided by two-photon excitation. Left panel, an image stack average (as in (*a*)). Right inset panels, examples of individual *X–Y* sections (section number reference is indicated; the effective optical width, approx. 1 µm) containing the soma; dotted line, a sampling segment (example) for the fluorescence brightness profile. Such samples were taken systematically in each *X–Y* section, normally by rotating the sampling segment in approximately 20° increments around the soma, throughout the Z-stack containing the soma. Plot, an example of 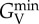 measurements in eight different *X–Y* sections of the same astrocyte (shown on the left): brightness profiles are normalized with respect to the brightness level inside the soma (see text) within each *X–Y* section; grey, individual profiles; black, average. Gap junctions are blocked with carbenoxolone (Material and methods).

Acute 350 µm slices from male Wistar rats (approx. eight-week-old) were transferred to the recording submersion chamber (Scientific Systems Design, NJ, USA) in an Olympus microscope integrated with a BioRad Radiance 2100 imaging system and coupled with a femtosecond-infrared laser (MaiTai, SpectraPhysics) and patch-clamp electrophysiology [23]. The superfusion solution contained (mM): 124 NaCl, 2 KCl, 2 CaCl_2_, 1 MgCl_2_, 10 glucose, bubbled with 95%/5% of O_2_/CO_2_. Astrocytes in dentate gyrus were held in whole-cell mode; the internal solution contained (mM): 135 K methanesulfonate, 10 HEPES, 10 Na_2_ phosphocreatine, 4 MgCl_2_, 4 NaATP, 0.4 NaGTP and 40 µM Alexa Fluor 594 (MW 759; chosen to ensure rapid morphological tracing of thin processes, and because it provides 100% staining of thin processes and even penetrates gap junctions). Pipette resistance was 3–4 MOhm, access resistance less than 15 MOhm. Passive astrocytes were identified electrophysiologically as non-excitable, low-input-resistance cells; this was subsequently verified by observing cell morphology (Results, figure 1). Dye escape through gap junctions was prevented by adding 100 µM carbenoxolone to the bath. The potential effect of carbenoxolone on fine glial morphology was tested in a homogeneous population of CA1 astrocytes: the mean tissue volume fraction (Results) in the control and carbenoxolone samples were, respectively, 0.0732 ± 0.0088 and 0.0728 ± 0.0096 (mean ± s.e.; *n* = 15 and 8; *p* > 0.95), indicating no effect of the drug. The laser beam intensity under the objective was less than 8 mW, to avoid any photobleaching of Alexa [23]. In each astrocyte, the emission intensity data were collected using a *Z*-series of thin two-photon excitation layers (approx. 1 µm thick; optical resolution in the *X–Y* plane was 0.4–0.6 µm). The original brightness levels were preserved throughout.

### (b) Tissue preparation and processing for electron microscopy

Animals were anaesthetized (urethane 1.5 g kg^–1^), perfused transcardially with 100 ml saline, followed by 100 ml 3.5% paraformaldehyde and 0.5% glutaraldehyde (0.1 M Na cacodylate buffer, pH 7.4) at room temperature (RT, 22°C). After perfusion, the brains were removed and coronal serial 50-μm sections were cut from each brain containing the whole dorsal portion of the hippocampus. Slices were immersed in 2.5% glutaraldehyde (0.1 M Na cacodylate buffer) for 24 h. The tissue was post-fixed with 1% OsO_4_ and 0.01% potassium dichromate (same buffer, 1–2 h, RT) dehydrated in graded solutions of ethanol (10 min each) and then 100% acetone (three changes 10 min each). Specimens were embedded in epoxy resin (Epon 812/AralditeM) as detailed earlier [25]. A total of 60–70 nm thick serial sections were cut (2–5% ethanol in water), allowed to form a ribbon on the bath surface and collected with Pioloform-coated slot copper grids. Sections were counterstained with saturated ethanolic uranyl acetate, followed by lead citrate, and were then placed in a rotating grid holder to allow uniform orientation of sections on adjacent grids in the electron microscope. Electron micrographs (6000×) were obtained in a JEOL 1010 electron microscope from the medial molecular layer of dentate gyrus, at a distance of 80–100 µm from the layer of neuronal cell bodies. Up to 100 serial sections per series were photographed. A cross-sectioned myelinated axon, mitochondria and dendrites spanning each section provided a fiduciary reference for initial alignment of serial sections.

### (c) Three-dimensional reconstruction and morphometry

Digital electron micrographs (1200 dpi) were aligned using SEM Align 1.26 and contours of individual dendritic spines, PSDs and astrocytic processes were traced digitally using IGL Trace 1.26 (http://www.synapses.clm.utexas.edu/). Section thickness was determined as described earlier [26] and was normally 60–70 nm (grey/white colour). Volumes and surface areas of individual structures were computed and three-dimensional (3D) objects were generated using the IGL Trace. Dendritic spines adjacent to identified astrocytic processes were reconstructed and analysed (see Results). Additional criteria for ‘mushroom’ spines were the presence of a spine apparatus and a complex PSD (perforated, U-shaped or segmented) whereas ‘thin’ spines had only macular PSDs and no spine apparatus [27,28].

For distance measurements, we used reconstructed surfaces represented by quasi-regular triangular lattices; lattice vertices provided a set of surface coordinates. Where required, centroids were calculated directly from such sets. Running through and measuring all distances between PSDs (all surface points) and the astrocyte membrane (all surface points with an upper distance limit) was carried out in 3D using the IGL Trace; the distance metric was Euclidean throughout. We exported 3D reconstructions to the 3D-Studio-Max 8 software for surface rendering. Statistica (StatSoft) was used for statistical testing. ANOVAs followed by Bonferroni's or Tukey's (unequal samples) tests were performed using OriginPro v. 7.5.

## 3. Results

### (a) Astrocytes in dentate gyrus: neuropil volume fraction in live tissue

To understand architectonics and space-filling features of individual astrocytes in dentate gyrus, we carried out single-cell experiments in acute hippocampal slices. We applied visual patch-clamp routines to identify individual astrocytes (occurring preferentially in the medial perforant path layer), filling them, in whole-cell mode, with the fluorescent tracer Alexa Fluor 594. To restrict indicator diffusion to individual cells, gap junctions were blocked by carbenoxolone (Materials and methods). Astrocytes were visualized in serial stacks of fluorescence images obtained with two-photon (2P) excitation of Alexa (figure 1*a*). Stable fluorescence was usually achieved within 5–10 min (access resistance typically approx. 10 MOhm). Pictures were taken about 20 min after entering whole-cell. All recorded cells were electrically passive: the average resting *V*_m_ and the input resistance were, respectively, −88.1 ± 0.7 mV and 5.9 ± 1.1 MOhm (mean ± s.e.m., *n* = 10; figure 1*b*; electronic supplementary material, figure S1), reflecting the properties of regular ‘passive’ astrocytes [29]. Cell morphology was consistent with that reported in the same area using Lucifer Yellow staining [24], including a proportion of astrocytes with long dendrite-like trunks (figure 1*a*). The average cross-sectional area occupied by an individual astrocyte arbour was 2631 ± 268 µm^2^ (*n* = 10); this corresponded to an average caliper size of 57.8 ± 18.5 µm, consistent with previous reports [30].

The tissue volume fraction occupied by glia, *G*_V_, should reflect basic properties of glutamate uptake in the area. *G*_V_ has hitherto been measured in fixed preparations using stereological rules [2,6]. To measure *G*_V_ in live tissue, we applied 2P microscopy: 2P excitation occurs entirely within the approximately 1 µm thick optical layer ensuring that no contaminating fluorescence is generated outside the focal plane. Because glial protrusions are normally much thinner than the translucent excitation layer, the sampled emission *F*(*i*,*j*) (*i* and *j,* pixel coordinates in the *X–Y* plane) is proportional to the local volume fraction of indicator-filled glia, or *G*_V_(*i*,*j*).

To translate *G*_V_(*i*,*j*) into the local volume fraction, we related it to the fluorescence *F*_max_(*i*,*j*) inside the dye-filled cell soma (i.e. 100% volume fraction) in the same focal plane (figure 1*c*): *G*_V_(*i*,*j*) = (*F*(*i*,*j*) − *F*_0_)/(*F*_max_ − *F*_0_) where *F*_0_ is background fluorescence (outside any stained structures). We used this approach to obtain two measurements. First, we excluded the areas occupied by the soma and thick (more than 1 µm) astrocyte processes. The resulting average value 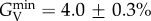 (*n* = 10 cells) thus reflected the tissue volume fraction occupied by the fine glial protrusions. Importantly, local 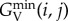 was relatively constant away from the soma (figure 1*c*), suggesting homogeneous glial coverage throughout the astrocyte domain. Second, we measured the *G*_V_ value that included all dendrite-like processes: *G*_V_ = 8.9 ± 0.7% (*n* = 10). This value should correspond to electron microscopy observations in which fragments of astrocyte processes are sampled arbitrarily. Because hippocampal astrocytes tend to occupy separate neuropil domains, with little or no spatial overlap [24,30,31], the value of *G*_V_ should therefore provide control for the completeness of astroglia reconstruction at the electron microscopy level.

### (b) Spatial juxtaposition of synapses and glia

**Figure 2.**
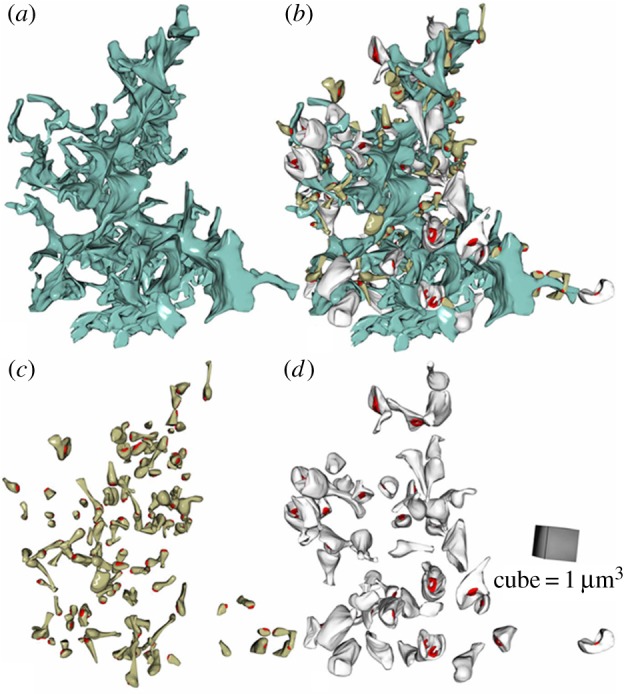
Three-dimensional reconstruction of contiguous astrocyte fragments with adjacent synapses. (*a*–*d*) A fragment of a dentate astrocyte reconstructed in three dimensions (blue, (*a*,*b*)) together with the adjacent dendritic spines (grey and dark yellow structures, (*b*)) equipped with PSDs (red). The spines are unambiguously separated into the sub-groups of thin (dark yellow, shown separately in (*c*)) and mushroom (grey, (*d*)). See the electronic supplementary material, figure S2, for examples of the original serial sections with identified structures.

In serial-section electron micrographs of medial molecular layer (Materials and methods), we identified glial fragments using the criteria described earlier [3,6,11,32] and further validated this by tracing contiguous structures across the series (figure 2*a*; electronic supplementary material, figure S2). Throughout the sample, the average tissue volume fraction occupied by glia was *G*_V_ = 9.18 ± 0.65% (*n* = 16 astrocyte fragments). This was in excellent agreement with *G*_V_ measured in live tissue (see above), indicating the accuracy of glial identification. We next reconstructed fragments of astrocytes together with the adjacent dendritic spines containing PSDs (figure 2*b*; electronic supplementary material, figure S2).

In 3D reconstructions, we could unambiguously distinguish between thin (*n* = 136) and mushroom (*n* = 53) dendritic spines (figure 2*c–d*), which differ more than fivefold in their average head volume [14,21,25]. Indeed, the average head volume for mushroom and thin spines was, respectively, 0.1998 ± 0.0156 and 0.0323 ± 0.0019 µm^3^ (mean ± s.e.m.). An average 3D nearest-neighbour distance between the PSD centroids among all spines was 0.57 ± 0.02 µm (*n* = 190). This was consistent with the volume density of excitatory synapses in dentate gyrus [33,34], and similar to the average nearest-neighbour inter-synaptic distance in area CA1 [10,35]. This consistency further validated the accuracy of reconstruction procedures.

**Figure 3.**
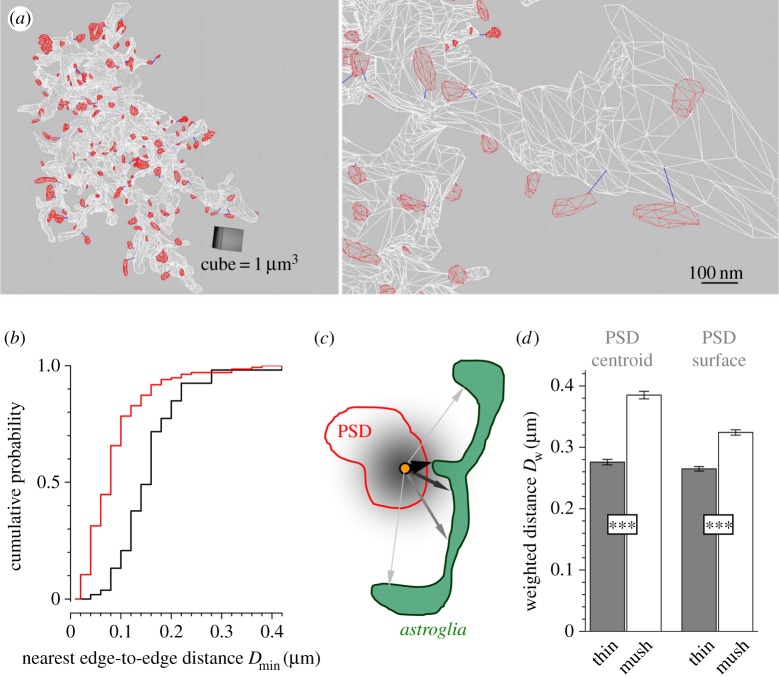
Astrocyte membranes are much closer to the PSDs occurring on thin, compared to mushroom, dendritic spines. (*a*) A diagram illustrating automatic measurement of the nearest edge-to-edge distances *D*_min_ between all individual PSDs (red mesh) and astrocyte membranes (white mesh) in space, at two levels of detail. Blue segments show *D*_min_ determined in 3D automatically; smooth surface rendering (as in figure 2) is omitted for clarity. (*b*) Distribution (cumulative probability plot) of *D*_min_ for individual PSDs occurring on thin (red, *n* = 136) and mushroom (black, *n* = 53) dendritic spines. (*c*) A two-dimensional diagram illustrating a rationale behind the weighting of PSD–glia distances. Distances between PSD (red, either the centroid only or all surface points) and all points on the surface of a neighbouring glial process (green) are measured, with larger distances bearing less weight (indicated by arrow thickness and shade of grey). The weighting reflects a rapid drop of transmitter concentration with distance from the site of release (yellow dot). See Results for details. (*d*) Grey and white columns, average (±s.e.m.) weighted PSD–astroglia distances *D*_w_ for thin and mushroom dendritic spines, respectively, measured with respect to either PSD centroids or all PSD surface points, as indicated (***p < 0.001).

First, we attempted to gauge glial coverage of synapses by calculating the proportion of synaptic circumvent approached by glia [10,11] (electronic supplementary material, figure S3). However, in some cases glial protrusions penetrated inside the synaptic apposition zone (electronic supplementary material, figure S4) whereas in other cases classifying the glia-circumvent ‘contact’ did not appear unambiguous (electronic supplementary material, figure S3). We therefore concentrated on an unsupervised methodology and calculated minimal (edge-to-edge) 3D distances between each individual PSD and the nearest astrocyte membrane, *D*_min_. To minimize bias associated with 3D-surface rendering, *D*_min_ was measured using non-smoothed coordinates (3.6 nm/pixel resolution; figure 3*a*). Perhaps unexpectedly, we found that the distributions of *D*_min_ for the sub-populations of mushroom and thin spines were markedly different (figure 3*b*): the average *D*_min_ values were, respectively, 145 ± 9 nm and 77 ± 6 nm (*n* = 53 and 156; *p* < 0.001). Glial protrusions occur therefore twice as close to the PSDs on thin dendritic spines in comparison to those on mushroom spines.

However, *D*_min_ does not necessarily represent the whole glial environment: for instance, closely approaching thin protrusions might connect to the bulk of distant glia. We therefore developed an integrative measure that would indicate how much glial surface how closely approaches each PSD. Because the closer glial surfaces are to the PSD the stronger impact they must have on any diffusion-mediated glia–synapse communication (figure 3*c*), we weighted all PSD–glia distances accordingly. The chosen weight function *w*(*r*) = *A*×exp(−18*r*^2^) approximated the drop of transmitter concentration at a distance *r* from the release site shortly post-release [6,9], with the factor *A* normalizing the sum of all weights as one: 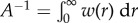. For each PSD, therefore, we calculated the average weighted PSD–glia distance 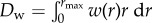 that incorporates information about all glial surfaces occurring at *r* < *r*_max_ from the PSD (*r*_max_ = 0.5 μm corresponded to the average distance to the next PSD).

The average *D*_w_ for mushroom and thin spines, respectively, was 0.385 ± 0.006 µm and 0.276 ± 0.005 µm (*p* < 0.001) when *D*_w_ was referred to the PSD centroid and 0.324 ± 0.004 µm and 0.264 ± 0.004 µm (*p* < 0.001) when *D*_w_ was referred to all points on the surface of each PSD. This result indicated that astroglia approached PSDs on thin dendritic spines more closely than PSDs on mushroom spines. (Additional calculations indicated that this difference was relatively insensitive to a particular form of *w*(*r*).)

## 4. Discussion

Here, we have analysed fine 3D architectonics of astrocyte compartments and adjacent excitatory synapses in the dentate neuropil. We have found that on average astroglia are significantly nearer to synapses on thin dendritic spines compared with those on mushroom spines. What could be the adaptive significance of this difference?

2P uncaging of glutamate at sub-synaptic resolution provided a map of AMPA receptor (AMPAR) distribution in dendritic spines visualized in hippocampal pyramidal neurons [16]. The authors showed that AMPARs are abundant (up to 150 per spine) in mushroom spines but are scarcely present in thin spines or filopodia of dendrites. This observation was consistent with the molecular machinery that controls, in a synergistic manner, both dendritic spine elongation and the pruning of local AMPARs in cultured cortical neurons [20]. In agreement with this, dendritic spines with larger, complex PSDs showed greater numbers of immuno-gold labelled AMPARs compared with spines with simple, macular PSDs in hippocampal neurons [17]. Remarkably, induction of long-term potentiation (LTP) by local glutamate uncaging could transform, in real time, thin spines deficient in AMPARs into the AMPAR-enriched large, mushroom-type spines [19]. Furthermore, classical electrophysiological observations propose that LTP may turn NMDA receptor-dominated (‘silent’) synapses into synapses relying on AMPAR-mediated transmission [36,37], evoking a concept of ‘learning spines’ as opposed to ‘memory spines’ [21,38].

This functional distinction between the two spine types may provide one possible explanation for tighter and snugger glial coverage around thin spines. In contrast to low-affinity AMPARs, high-affinity synaptic NMDA receptors could in some conditions sense glutamate released outside the immediate synapse [7,9]. This ‘spill-in’ signal is controlled by high-affinity glial glutamate transporters [2,4,39], which could thus be particularly important for synapses that rely on high-affinity (NMDA) receptors.

The important adaptive role for the extent of synaptic glial coverage could also arise from the findings that hippocampal astrocytes can exchange transmitter signals with the neighbouring neurons [40,41]. Because such signalling relies on rapid extracellular diffusion outside the synaptic cleft, changes in the spatial juxtaposition of synapses and glia should have a direct impact on this mode of neuron–glia communication [12,13].

## Supplementary Material

Supplementary figures
