# Supplementary material for: Glia selectively approach synapses on thin dendritic spines
Source: Philos Trans R Soc Lond B Biol Sci. 2014 Oct 19;369(1654):20140047. doi: 10.1098/rstb.2014.0047 (PMC4173297; doi:10.1098/rstb.2014.0047)
Supplement: Supplementary figures [file rstb20140047supp1.pdf]

Supplemental Material

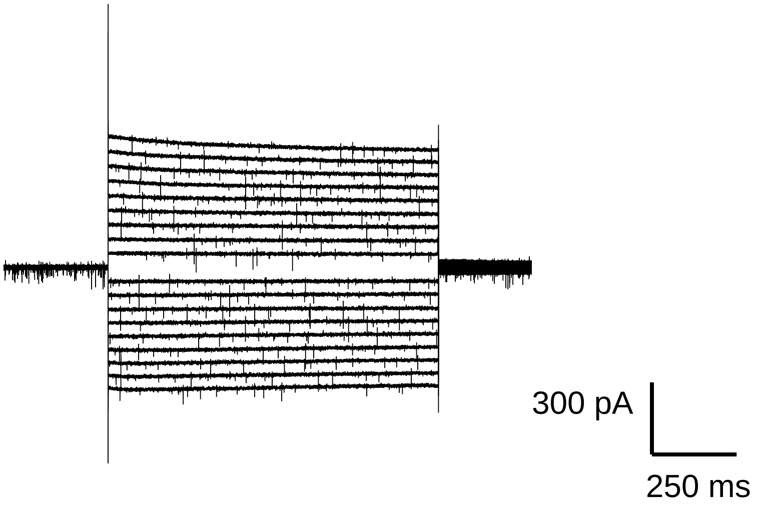

Figure S1. A characteristic current-voltage relationship for recorded passive astocytes.

Whole-cell voltage-clamp current responses to voltage steps (shown in 1 mV increments; 1 second duration) are depicted. The data show a linear voltage-current relationship indicating a passive astrocyte.

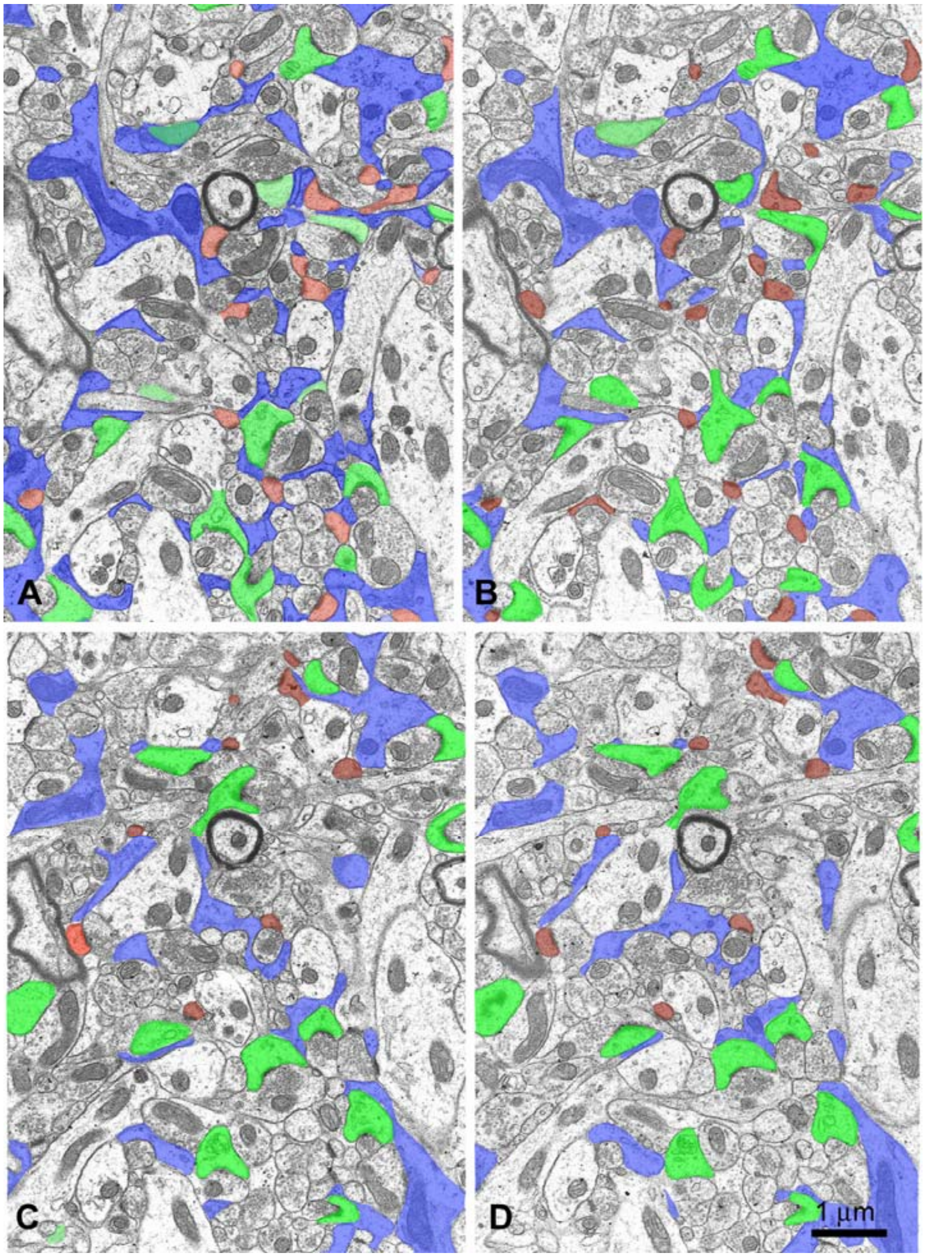

Figure S2. An example showing electron micrographs of four consecutive images in which synaptic structures (brown and green colors show dendritic spine profiles for thin and mushroom spines,correspondingly) and glial fragments (blue) are identified for three-dimensional reconstructions.

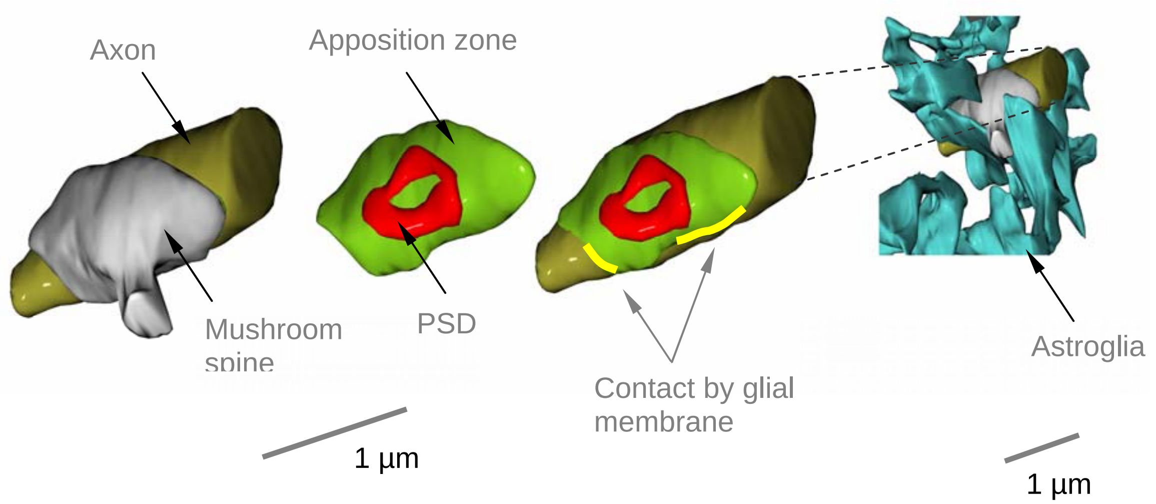

Figure S3. An example showing the approach of synaptic circumventing by astroglial membranes.

Three-dimensional reconstruction of an axonal fragment synapsing on a mushroom spine. Notations self-explanatory. The candidate glia-circumventing contact areas depicted in yellow might depend on the surface-rendering algorithm and are therefore potentially biased by the size of the adjacent synaptic strictures. In addition, it was sometimes difficult to say to what extent glial membranes should approach the synaptic circumventing for it to be classified as "contact".

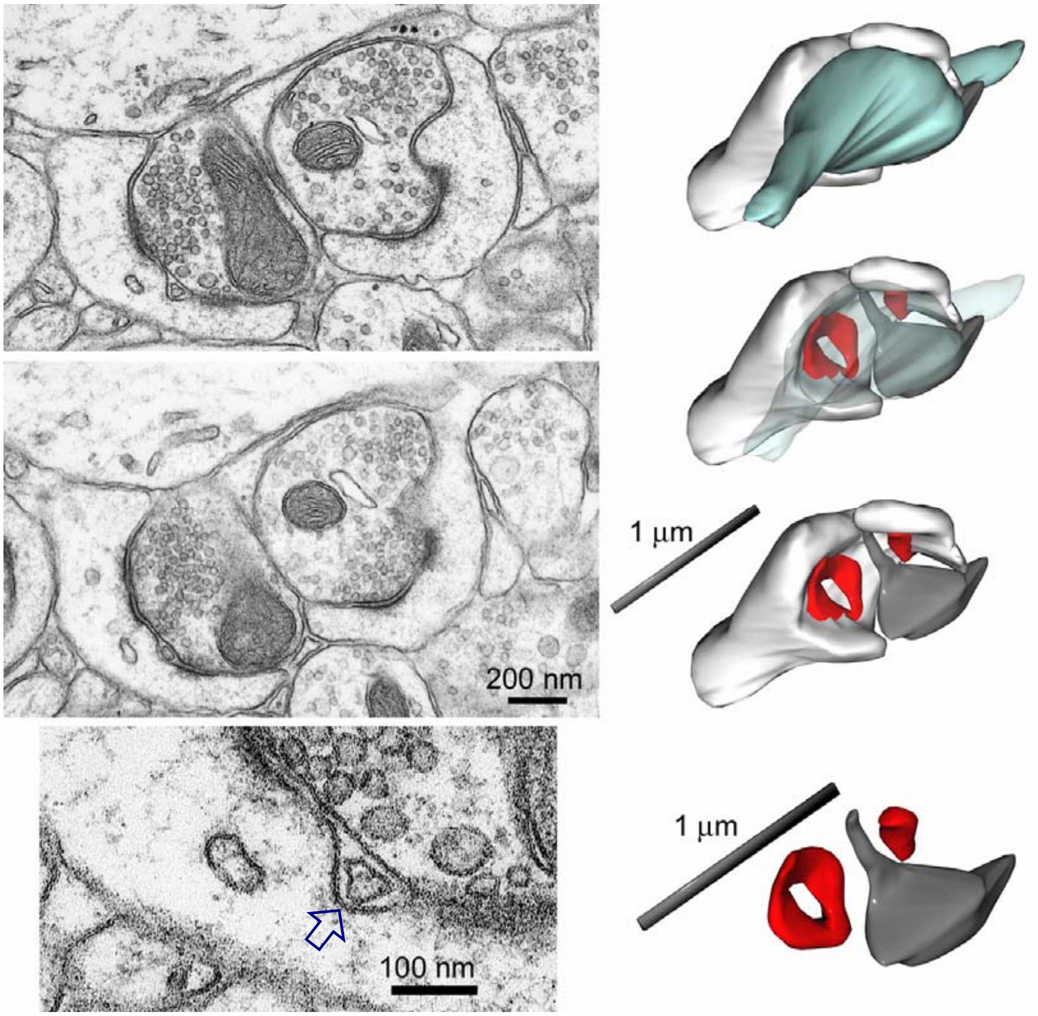

Figure S4. An example illustrating penetration of astrocyte protrusions into the synaptic apposition zone.

Left, electron micrographs of serial sections through a mushroom spine with a perforated and segmented postsynaptic density (PSD); bottom image, arrow indicates an astrocyte fragment protruding into the space between PSD segments. Right, 3D reconstruction of the spine (gray), PSD (red) and glial fragment (green) showing that the surface rendering algorithm preserved the original 3D arrangement. Notations are self-explanatory.
